# Grouping strategies in number estimation extend the subitizing range

**DOI:** 10.1038/s41598-020-71871-5

**Published:** 2020-09-11

**Authors:** Paula A. Maldonado Moscoso, Elisa Castaldi, David C. Burr, Roberto Arrighi, Giovanni Anobile

**Affiliations:** 1 Department of Neuroscience, Psychology, Pharmacology and Child Health, University of Florence, Florence, Italy; 2 Institut für Psychologie, Universität Regensburg, Regensburg, Germany; 3 Department of Translational Research and New Technologies in Medicine and Surgery, University of Pisa, Pisa, Italy

**Keywords:** Attention, Perception

## Abstract

When asked to estimate the number of items in a visual array, educated adults and children are more precise and rapid if the items are clustered into small subgroups rather than randomly distributed. This phenomenon, termed “groupitizing”, is thought to rely on the recruitment of the *subitizing* system (dedicated to the perception of very small numbers), with the aid of simple arithmetical calculations. The aim of current study is to verify whether the advantage for clustered stimuli does rely on subitizing, by manipulating attention, known to strongly affect attention. Participants estimated the numerosity of grouped or ungrouped arrays in condition of full attention or while attention was diverted with a dual-task. Depriving visual attention strongly decreased estimation precision of grouped but not of ungrouped arrays, as well as increasing the tendency for numerosity estimation to regress towards the mean. Additional explorative analyses suggested that calculation skills correlated with the estimation precision of grouped, but not of ungrouped, arrays. The results suggest that groupitizing is an attention-based process that leverages on the subitizing system. They also suggest that measuring numerosity estimation thresholds with grouped stimuli may be a sensitive correlate of math abilities.

## Introduction

Humans can generally count or estimate the number of objects in a scene quite easily, yet the perceptual mechanisms and the cognitive strategies underlying this ability are still little understood. Numerical judgments are extremely fast and virtually errorless up to four items, while they become slower or more approximate for larger numerosities^1–3^. This behavior suggests the existence of two independent systems for perception of very small and larger numerosities, the subitizing and the Approximate Number System (ANS) respectively^4^.

Interestingly, counting speed of larger numerosities also increases considerably if stimuli are grouped into smaller clusters^5,6^, a phenomenon that has been termed *groupitizing*^7^. Counting is particularly fast when the number of clusters and the number of items included in each cluster is very low (e.g. 8 = 4 + 4), falling within the subitizing range^7^. Two recent studies have generalized the groupitizing effect to non-spatial grouping cues, different numerosity tasks and formats. Ciccione and Dehaene^8^ showed a groupitizing advantage only when items were divided into clusters of the same number of items, irrespective whether the items were grouped spatially or by color alone. Anobile et al.^9^ went on to show that groupitizing can also boost sensory precision measured with an approximate numerosity estimation task, both for spatial arrays and temporal sequences. Starkey and McCandliss^7^ noticed that school-age children with higher arithmetical abilities took most advantage of groupitizing cues, while there was no groupitizing effect in preschoolers, suggesting that the ability to groupitize may reflect the use of arithmetical strategies (e.g. divide-and-sum).

A reasonable conclusion from these studies is that groupitizing arises from two independent factors: the ability to subitize small groups parsed from the larger set, and the ability to combine the group estimates through mental calculation. The first aspect implies that participants may recruit the subitizing system to estimate numerosities higher than the normal 4-item limit. This strategy would require considerable cross-talk between subitizing and ANS, usually considered to be independent systems. However, there is some evidence for interconnection between the systems. Under dual task conditions, sensory thresholds for estimating numerosities in the subitizing range become comparable to those measured in the estimation range, suggesting that the estimation system works even within the subitizing range, but performance for low numbers normally augmented by the automatic deployment of visuo-spatial attentional resources^10–12^. The heavy reliance of subitizing on attention may therefore constitute a characteristic feature of this system and explain its higher precision. Thus, measuring performance under conditions of deprived attention may serve as a diagnostic test of whether groupitizing is based on the subitizing system.

Number estimation is not always veridical. The clearest example comes from *numberline* studies, which require participants to map number onto space. Under many conditions, including deprived attention, the mapping shows a strong compressive non-linearity^13^. While this has been described as reflecting a native logarithmic system of encoding number^13^ several recent studies explain the non-linearity as an example of “central tendency” or “regression to the mean”, a principle observed in almost all perceptual systems^14^. Regression to the mean is well described within the Bayesian framework, where the mean can be considered a Bayesian *prior*^13,15,16^. An important prediction from this approach is that the magnitude of the compressive non-linearity should vary with the precision of the numerosity judgments: the worse the precision (higher Weber fractions), the greater should be the non-linearity. If groupitizing is rooted in the subitizing system, which needs attention to boost precision^10^, we expect there to be less regression to the mean for grouped than ungrouped stimuli, and that this advantage should disappear under attentional deprivation.

In the current study we tested whether the grouping-induced improvements in precision and accuracy of number estimation is based on extending the subitizing system to larger numerosities. To this aim we measured precision and accuracy of numerosity estimation for grouped and ungrouped arrays while modulating attentional resources with dual tasks. If the groupitizing phenomenon is rooted in the subitizing system, attentional deprivation should affect precision more for grouped than ungrouped stimuli. We further explored whether groupitizing may rely on arithmetical computation, with a preliminary study correlating simple calculations skills with precision for estimating grouped or ungrouped numerosities.

## Methods

### Power analyses

Sample size was calculated with a Power analyses using G*Power software (version 3.1). As the main goal of the current experiment was to detect a numerosity thresholds change under attentional load the analyses aimed to calculate the required sample size to reliably detect a difference between two dependent means: average Weber Fractions in single and dual task conditions (two tailed paired t-test). The effect size was estimated from Burr et al.^10^. With an ⍺ = 0.05 and a Power of 0.95, the analyses suggested a required sample size of 6.

### Participants

Twelve young adults (mean age = 26.1, standard deviation = 2.9, range = 22–32) participated in this study. Participants were all psychology students with no mathematical or other learning disorders nor over-exercised calculation skills and all with a normal or corrected-to-normal vision.

### Materials and procedure

Stimuli were generated and presented with PsychToolbox^17^ routines for Matlab (ver. R2016b. 9.1.0.441655. The Mathworks, Inc., https://it.mathworks.com). Subjects sat 57 cm from a 19″ screen monitor (60 Hz), in a quiet and dimly light room. One experimenter (P.A.M.M.) performed the tests throughout the study. The experimental procedures were approved by the local ethics committee (*Comitato Etico Pediatrico Regionale—Azienda Ospedaliero-Universitaria Meyer*, Florence). The research was performed in accordance with the Declaration of Helsinki and informed consents were obtained from all participants prior to the experiment.

Participants each performed five sessions: in four they were asked to estimate the numerosity of ungrouped or grouped arrays both in single or dual task conditions, while in the fifth session they were given a mental calculation task. The conditions were tested separately with the order counterbalanced across subjects. No feedback was provided, and participants were not informed about the numerosity range. They were also not informed about the different spatial structures of the numerical arrays (ungrouped or grouped), and they were left free to choose any strategy to solve the task, and the possibility of performing mental calculation with the grouped stimuli was never mentioned.

### Numerosity stimuli and experimental paradigm

Stimuli were the same as those used by Anobile et al.^9^. The arrays were sets of white squares (0.4° × 0.4°) with black borders (in order to balance overall luminance) constrained within a square area of 6° × 6°. The only difference from Anobile et al.^9^ was that in each trial, one item was randomly selected and replaced with a different shape, either a diamond, a triangle or a circle (with a total area equal to that covered by the squares).

**Figure 1:**
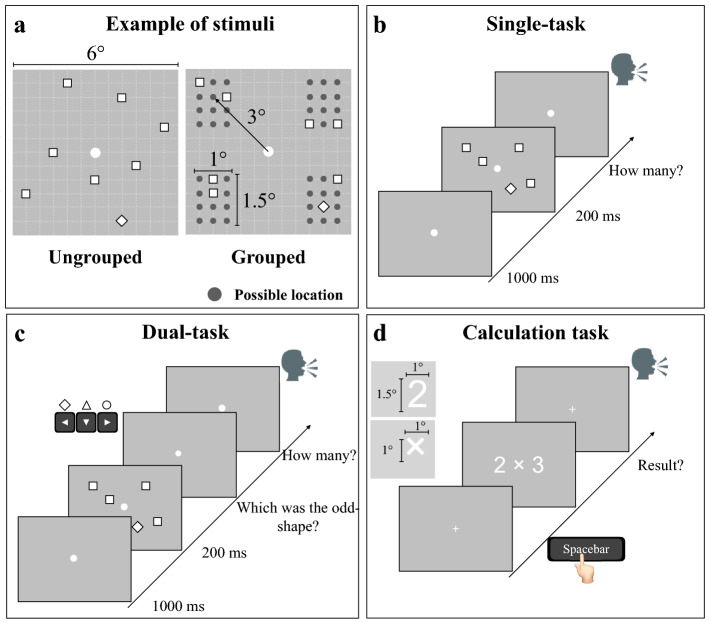
Illustration of the stimuli and procedure. (**a**) Illustration of the procedures followed to generate the stimuli in the ungrouped and grouped conditions. (**b**,**c**) In the numerosity estimation tasks each trial started with a central fixation point, followed by a briefly flashed ensemble of squared items, with one differing shape (diamond in the example). (**b**) Single-task: Participants were asked to ignore the odd-shaped item and to verbally report the perceived numerosity. (**c**) Dual-task: participants first classified the odd-shape (by appropriate keypress), then verbally reported the perceived numerosity. (**d**) The calculation task started as the participant pressed the spacebar. On every trial, a particular arithmetical operation appeared on the screen (lasting until the response), and participants verbally reported (as fast as possible) the result.

In the ungrouped conditions, the position of each item was randomly selected from 106 possible positions within the stimulus area, the centers of equally spread sectors within the 6 × 6 area (each grid 0.5° × 0.5°). For the spatially grouped condition, items were arranged within a maximum of 4 groups (Fig. 1). Each group (spanning over a max area of 1 × 1.5°) was located in one quadrant centered at 3° from the central fixation point. Each group was randomly assigned to one quadrant (between 1 and 4), then the individual items positions were randomly selected out the 12 possible locations in the selected quadrant. Within each quadrant, the maximum center-to-center distance between elements was 2° and the minimum was 0.5°.

Each trial started with a black central fixation point that turned white after 1 s and remained on screen for the entire experiment. After another 1 s an array of items was centrally displayed for 200 ms, followed by a blank screen. In the single tasks (performed separately with ungrouped and grouped stimuli), participants were asked to verbally estimate the numerosity of the array, disregarding the shape of the individual items. The response was entered by the experimenter on the numeric keypad, who also initiated the following trial. Participants were asked to respond quickly, but to concentrate on accuracy. In the dual-tasks (again, performed separately with ungrouped and grouped stimuli) participants were asked first to identify the oddly shaped item by pressing the appropriate arrow key (diamond: left arrow; triangle: down arrow; circle: right arrow), then to verbally estimate the numerosity of the array. The experimenter (blind to the stimuli) hit the spacebar as soon as the response was spelled out, then inserted the number on a numeric pad.

We tested all numerosities between 5 to 17. In the grouped conditions, each numerosity was organized into 2–4 clusters, each comprising a variable number of items (between 2 and 6), resulting in the following configurations: 2, 2, 1–3, 3–3, 3, 1–2, 2, 2, 2–4, 4–3, 3, 3–3, 3, 3, 1–3, 3, 3, 2–3, 3, 3, 3–4, 4, 4–5, 5, 3–4, 4, 3, 3–4, 4, 4, 3–4, 4, 4, 4–5, 5, 6–5, 4, 4, 4. All clusters except three (13 = 5, 5, 3; 16 = 5, 5, 6; 17 = 5, 4, 4, 4) contained 1 to 4 elements.

On every trial, numerosities and configuration patterns (i.e. 3,3,3,1 or 3,1,3,3) were randomly selected. Each participant completed 150 trials for each condition, with each numerosity presented in mean 12 times, for a total of 600 trials for the entire experiment. Trials with response times higher than 3 standard deviations were considered outliers and eliminated from the analysis (0.8% of the trails).

### Mental calculation test

Mental calculation proficiency was measured by a custom-made computerized test. Each trial started with a central fixation cross. As soon as the participants pressed the space bar, the stimuli (1° × 1.5° digits, and 1° × 1° operand, Arial font) were displayed. Each trial required the participant to mentally solve an arithmetic operation. Each participant solved 37 operations in total. Each operation was randomly selected trial-by-trial between: 3 + 3, 4 + 2, 2 + 5, 3 + 4, 4 + 4, 5 + 3, 3 + 6, 4 + 5, 2 × 3, 2 × 4, 2 × 5, 2 × 6, 2 × 7, 2 × 8, 2 × 9, 3 × 3, 3 × 4, 3 × 5, 3 × 6, 4 × 4, 4 × 5, 4 × 6, 6–3, 6–4, 7–3, 7–5, 8–3, 8–4, 9–4, 9–6, 2 + 1 + 2, 3 + 1 + 3, 3 + 3 + 3, 3 + 4 + 4, 5 + 3 + 5, 5 + 6 + 5, 6 + 5 + 6. Participants mentally calculated the result as fast as possible and responded verbally (no explicit time limit was provided). The experimenter (blind to the stimuli) hit the spacebar as soon as the participants spelled out the result (which recording response time), then entered the response on the numeric keypad. Trials with response time higher than 3 standard deviations were considered outliers and eliminated from the analysis (1.3% of trails).

### Data analysis

Data were separately analyzed for each subject. For the numerosity estimation task we calculated the average perceived numerosity (accuracy) and the response standard deviation (precision), separately for each numerosity and condition. Standard deviations were divided by the corresponding perceived numerosity, resulting in the Weber fraction (Wf), a dimensionless index of precision^18^. The Weber fractions calculated for each separate numerosity were also averaged across numerosity levels, in order to obtain a summary precision index.

The magnitude of the attentional cost induced by grouped spatial structure was measured as the normalized difference between average Weber fractions calculated in the single (ST) and dual (DT) tasks, averaged across numerosity levels:

1 $$Attentional\;cost= \frac{{Wf}_{DT}- {Wf}_{ST}}{{Wf}_{DT}+{Wf}_{ST}}$$

where ${Wf}_{DT}$ and ${Wf}_{ST}$ are average Weber fractions for the dual and single tasks.

The thresholds improvements induced by grouping in the single task was measured as the normalized difference between average Weber fractions calculated in the ungrouped (NG) and grouped (G) conditions, averaged across numerosity levels:

2 $$Groupitizing \;advantage= \frac{{Wf}_{NG}- {Wf}_{G}}{{Wf}_{NG}+{Wf}_{G}}$$

where ${Wf}_{NG}$ and ${Wf}_{G}$ are the average Weber fraction for the ungrouped and the grouped conditions in the single task.

Weber fractions were analyzed with Repeated Measures ANOVA and Bonferroni corrected post-hoc t-tests. Effect sizes (η^2^ and Cohen’s d) are also reported when appropriate. The relation between attentional cost, total numerosity and number of groups was analyzed with zero-order (Spearman) and partial correlations. Log_10_ Bayes factors (LogBF) are reported alongside standard Rho (ρ_s_) and p-values. Positive Log_10_ Bayes factors should be interpreted as lending substantial (0.5–1), strong (1–1.5), very strong (1.5–2) and decisive (> 2) support to the alternative hypothesis. Negative LogBF within these ranges is evidence for the null hypothesis.

To evaluate non-linear compression of mean estimates of numerosity we fitted the data with power functions:

3 $$y=a{N}^{b}$$

where *y* is the average estimate of numerosity, *N* physical numerosity and *a* and *b* constants free to vary. The value of the exponent *b* is an index of non-linearity, with *b* = 1 implying a linear relationship, and *b* < 1 a compressive non-linearity (*b* = 0.5 implies square root).

The Bayesian central tendency model assumed that the perceived numerosity *y* was given as a weighted average of the physical numerosity and the mean of the range.

4 $$y=N\left(1-{w}_{p}\right)+{w}_{p}\stackrel{-}{N}$$

where ${w}_{p}$ is the weight assigned to the prior, which for an optimal observer is proportional to the relative reliabilities (inverse variances) of the two sources of information. Under the simplifying assumption of Weber’s Law, this becomes:

5 $${w}_{p}= \frac{{\left({Wf}_{i}\cdot N\right)}^{2}}{{\left({Wf}_{i}\cdot N\right)}^{2}+{\sigma }_{P}^{2}}$$

where ${Wf}_{i}$ is the Weber fraction for condition, and ${\sigma }_{P}^{2}$ is the variance of the prior, estimated to best fit all four conditions simultaneously.

For the mental calculation task, two separate *z* scores were calculated for each participant (using the mean and the standard deviation of the entire group), one for accuracy, the other for response speed. We then averaged the two *z* scores to yield a combined math performance index, following the procedure previously used by Anobile et al.^18^. Participants were categorized as belonging to the “low” or “high” math sample if the combined z-score for mental calculation was below or above the 50th percentile. To evaluate the relation between numerosity estimation and calculation skills we performed standard Pearson’ correlations, with correction for multiple comparisons.

Statistical analyses were performed using JASP (version 0.12.2, The JASP Team 2020, https://jasp-stats.org) and Matlab (R2016b).

## Results

### Effect of grouping and attention on numerosity estimation thresholds

**Figure 2:**
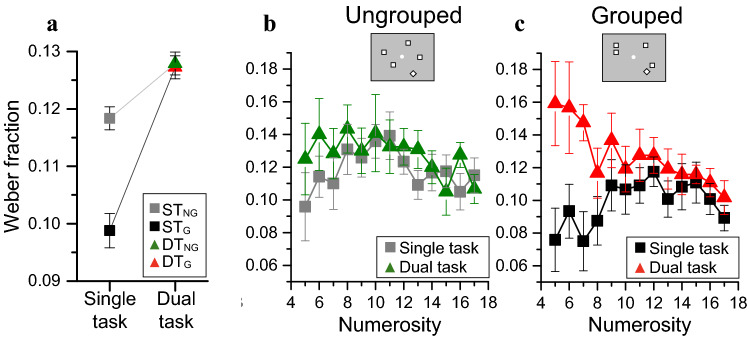
Effect of attention on numerosity estimation precision. (**a**) Average Weber fractions for the four conditions showing the interaction between attentional load and stimulus configuration on numerosity estimation. The average Weber fraction for the 4 conditions were: ST_NG_ = 0.118 ± 0.002; ST_G_ = 0.099 ± 0.003; DT_NG_ = 0.129 ± 0.002; DT_G_ = 0.126 ± 0.002. (**b**,**c**) Average Weber fractions as a function of numerosity plotted separately for ungrouped (**b**) and grouped stimuli (**c**) for the single (squares) and dual (triangles) tasks. Symbols refer to average responses, with error bars ± 1 sem.

We used a dual-task paradigm to measure the effect of attentional deprivation on precision and accuracy of numerosity estimation for ungrouped and grouped spatial arrays. Participants estimated numerosity, either during a concurrent visual search task (spot out the odd-shaped item), or with the visual distractor present, but ignored (single-task). Figure 2a shows that when the distractor was ignored, leaving attentional resources for the numerosity task, there was a strong groupitizing advantage, about 20% on average. Depriving attention affected grouped but not ungrouped stimuli, annulling the groupitizing advantage. For ungrouped stimuli the small effect of attentional deprivation was similar at all numerosities (Fig. 2b), while for grouped stimuli, it was clearly strongest at lower numerosities (Fig. 2c).

**Figure 3:**
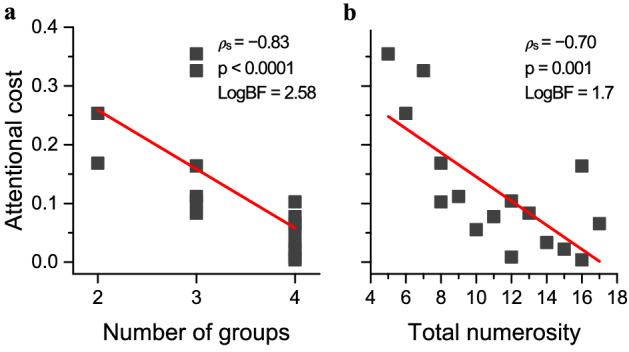
Relationship between attentional cost, number of groups and total numerosity. Attentional cost correlated with the number of groups (**a**) and with the total numerosity (**b**).

These effects were born out by three-way repeated measures ANOVA, with spatial structure (ungrouped or grouped), attentional load (single or double task) and numerosity (13 levels) as factors. There were significant main effects for spatial structure (F_(1,11)_ = 5.8, p = 0.034, η^2^ = 0.013, d = 0.23) and for attentional load (F_(1,11)_ = 11.2, p = 0.006, η^2^ = 0.046, d = 0.44). Crucially, the interaction shown in Fig. 2a between attentional load and spatial structure was significant (F_(1,11)_ = 5.4, p = 0.04, η^2^ = 0.011, d = 0.21). Post-hoc tests showed that with full attention, Weber fractions for grouped arrays were significantly lower than those for ungrouped arrays (t = 3.35, p_bonf_ = 0.017, squares in Fig. 2a), while in the dual-task they were statistically indistinguishable (t = 0.11, p_bonf_ = 1). Modulating attention did not alter Weber fractions for ungrouped arrays (t = 1.37, p_bonf_ = 1) while for grouped arrays, Weber fractions in dual-task were higher than that in single-task (t = 4.082, p_bonf_ = 0.004). There was also a significant interaction between numerosity and attentional load, being stronger at low numerosities (F_(12,132)_ = 3.14, p < 0.0001, η^2^ = 0.04, d = 0.41). The triple interaction was not significant (F_(12,132)_ = 0.9, p = 0.58, η^2^ = 0.012, d = 0.22). Yet, if groupitizing is based on a capacity-limited, subitizing-like system, depriving attention should most strongly impact the lowest grouped numerosities. Indeed, although the triple interaction did not reach significance, attention seems to affect estimation thresholds more for low numerosities, and only for grouped stimuli. Planned comparison t-tests confirmed that attentional deprivation did not significantly affect estimation thresholds of ungrouped stimuli for any of the numerosities tested (all p > 0.05 Fig. 2b). On the other hand, when the stimuli were spatially grouped, attention most strongly modulated estimation thresholds for the lowest numerosity (N5: t = 5.149, p_bonf_ = 0.0007; N6: t = 3.913, p_bonf_ = 0.158; N7: t = 4.48, p_bonf_ = 0.015; p_bonf_ > 0.05 for all the other numerosity, Fig. 2c, see also Fig. 3b.

To avoid a systematic association between total numerosity and number of groups, numerosities in the grouped condition were presented with different configurations, varying between 2 and 4 clusters. For example, the number eight was shown either with the (2, 2, 2, 2) or with the (4, 4) configurations. We tested whether the attentional modulation of thresholds was particularly marked for certain configurations, and whether it depended primarily on the number of groups or on the total numerosity, or both. We correlated the attentional cost (defined as the normalized difference between Weber fractions in the single and dual conditions: Eq. 1) with the number of groups and total numerosity (Fig. 3). As larger numerosities were generally divided into more groups than lower numerosities (positive correlation between total numerosity and number of subgroups: ρ_s_ = 0.51, p = 0.02, LogBF = 0.8), we also calculated partial correlations, evaluating the variance independently explained by each of these factors (total numerosity or number of groups). Attentional cost negatively correlated with both the number of groups and total numerosity (both ρ_s_ < 0.001, LogBF > 1.7), suggesting that the detrimental effect of attention was higher when both the number of groups and the total numerosity were lower and tended to decrease for larger numerosities. The correlation between the attentional cost and total numerosity remained significant even when taking into account the effect of the number of groups (ρ_s_ =  − 0.53, p = 0.017, LogBF = 0.90). Similarly, the correlation between attentional cost and number of groups also remained significant when controlling for the total numerosity (ρ_s_ =  − 0.62, p = 0.006, LogBF = 0.99). These results indicate that attentional deprivation acts on both the total numerosity and on the number of groups: its negative impact on estimation thresholds was strongest for the lowest numerosities and for stimuli divided into fewer groups.

### Effect of spatial structure and attention on accuracy of estimating numerosity

Under many conditions, including deprived attention, the mapping shows a strong compressive non-linearity^13^, considered by many as an example of regression to the mean. If groupitizing is rooted in the attention-dependent subitizing system, which requires attention to boost numerical estimation precision, the effects of grouping and attentional deprivation should also be evident in estimation accuracy.

**Figure 4:**
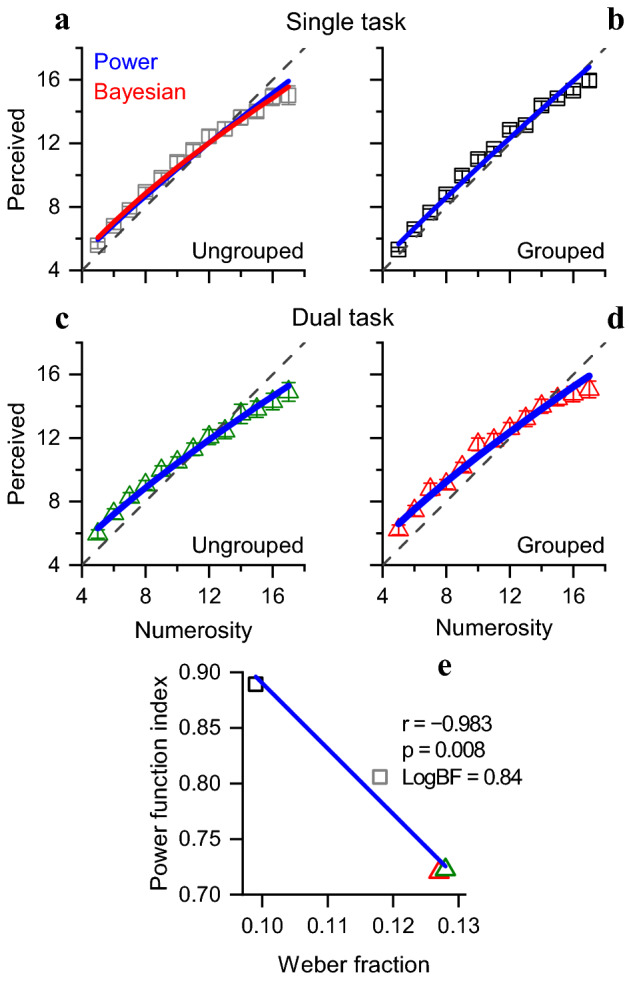
Perceived numerosity. Perceived numerosity as a function of physical numerosity for estimation of ungrouped (**a**) or grouped (**b**) stimuli in single and dual task (**c**,**d**). Continuous lines are the best fit of power function (blue) and Bayesian modelling (red). (**e**) Power function index correlated with the average Weber fraction. Symbols refer to average across participants, with error bars ± 1 sem.

Figure 4a–d shows the average estimates of numerosity for the four conditions. In general, low numerosities were overestimated and high numerosities underestimated, both following a regression to the mean. However, as usually observed, the regression to the mean was greater at high numerosities (where precision is less), resulting in a strong compressive non-linearity. To measure the non-linearity created by these biases, we fitted each set of data with a power function (Eq. 3, methods), shown by the blue lines. The fits were all very good (total R^2^ over all conditions = 0.986).

Importantly, as predicted, the non-linearity was not the same in all four conditions, but was highest for conditions with the highest Weber fractions. Figure 4e plots the index of the power function against average Weber fraction. The non-linearity clearly increases with Weber fractions, from 0.89 for the grouped single task condition (index of 1 means a linear function), to 0.80 for the ungrouped single task condition to 0.72 for the two dual task conditions. Where performance is most precise, it is also most accurate. The correlation between the two measures was r =  − 0.983, p = 0.008, LogBF = 0.84.

To test the quantitative predictive power of the Bayesian model of central tendency, we fitted the data with the Bayesian prediction, given by Eq. (4) of methods. The equation essentially states that perceived numerosity will be a weighted average of the actual physical numerosity of the stimulus and the mean numerosity of the range tested (the *prior*). Relative weighting of the two is determined by their precision: the more precise the estimates, the higher the weighting Eq. (5). That has two consequences. Assuming constant Weber fractions implies that thresholds increase linearly with numerosity, so the regression effects will be more pronounced at higher than at lower numerosities, leading to the compressive non-linearity. Secondly, as the Weber fractions increase between conditions, the prior (which we assume to remain constant between conditions) will have greater effect, resulting in the greater non-linearities that we observe (Fig. 4e).

The fits are shown by the red curves of Fig. 4a–d. The four fits have only 1 degree of freedom for all of them, the width of the prior (${\sigma }_{P}$ of Eq. 5) was constant for all four conditions, selected to simultaneously minimize the residuals of all four fits. The resulting fits were excellent, with total R^2^ = 0.988 (compared with 0.986 for the power fits). Thus, the Bayesian central tendency model explains well the data, qualitatively and quantitatively.

### Relation with arithmetical abilities

Despite the relatively small number of participants in this study (primarily designed to examine in detail the effects of attention on groupitizing), we also looked for possible correlations between groupitizing and math skills. Participants did a simple speeded calculation test described in methods, which was scored for both speed and accuracy. The average accuracy across participants was 90% ± 7%, and average speed was 1.3 ± 0.3 s. We combined z-scores of speed and accuracy (see methods) and correlated this index against Weber fractions for ungrouped and grouped stimuli.

**Figure 5:**
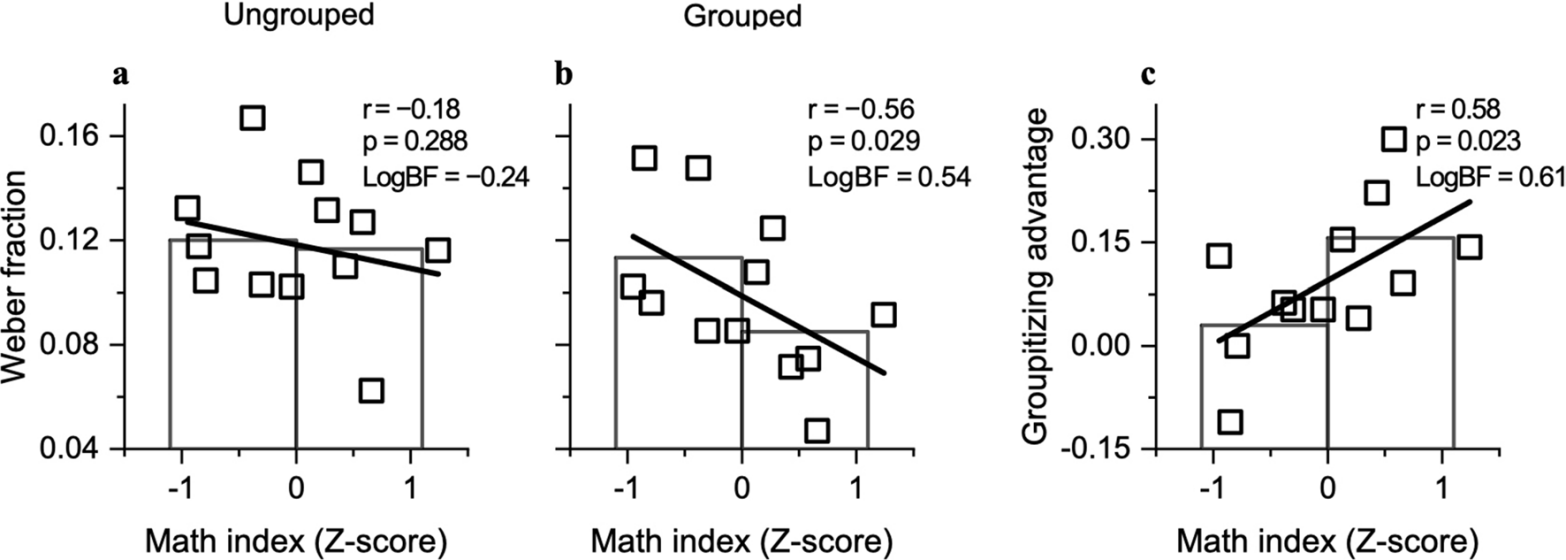
Relation between estimation precision and mental calculation abilities. (**a**,**b**) Weber Fractions plotted against math index for all participants. Bars show averages for median split. The correlation was insignificant for ungrouped but significant for grouped stimuli (values in graph and main text). (**c**) Groupitizing advantage as a function of math index. The correlation was positive and significant (values in graph and main text).

For ungrouped stimuli, Weber fractions were uncorrelated with the math index (r =  − 0.18, p = 0.288, LogBF =  − 0.24; Fig. 5a); but for grouped stimuli the correlation was significant, and remained close to significance after correcting for multiple comparison (α = 0.5/2: r =  − 0.56, p = 0.029, LogBF = 0.54; Fig. 5b). We also found that participants with higher arithmetical skills gained more from grouping of stimuli than less skilled participants (r = 0.58, p = 0.023, LogBF = 0.61; Fig. 5c). While these results should be taken with caution before replication in future studies, they suggest the very interesting possibility that groupitizing could be a sensitive predictor of math skills.

## Discussion

The aim of the present study was to directly test whether the *groupitizing* phenomenon^7^ depends on subitizing, by measuring the consequences of depriving attentional resources on numerosity estimation thresholds of spatially grouped and ungrouped items. As previous studies^9^ have shown, numerosity thresholds for spatially grouped stimuli were lower than for randomly scattered stimuli. However, depriving attention with a concomitant dual task completely obliterated the groupitizing advantage, consistent with the suggestion that it relies on subitizing. We also explored the link between groupitizing and arithmetic, and showed that simple mental calculations skills in adult participants correlated with estimation thresholds for grouped but not ungrouped stimuli, and also with the advantage given by grouping.

Although subitizing was originally thought to be pre-attentive, dependence on attention has become a signature of the subitizing system. Many studies have shown that attention has a much stronger detrimental effect in the subitizing than estimation range, enough to equate subitizing precision and reaction times to those of higher numerosities during dual tasks^10,12,19–21^. The selective detrimental effect of attentional deprivation in the subitizing range was reinforced by a recent clinical single case study with a simultanagnosic patient (PA)^22^, who suffered a severe visual attentional deficit. PA showed no subitizing advantage for low numerosities, while his numerosity perception was relatively spared for intermediate numerosities, above the subitizing range. The subitizing advantage, at least in the visual domain, could thus emerge from the well-known capacity-limited attentive tracking system, that allows precise tagging of a few objects in space^23^. Other studies show that depriving auditory and haptic attentional resources also affects visual subitizing^19^. Future studies should investigate the effect of cross-modal attention deprivation on groupitizing.

The current study showed that performing a dual task completely eliminates the groupitizing advantage for estimation thresholds, in the same way that it eliminates the subitizing advantage for low numbers: estimation thresholds for grouped arrays in dual task became like those measured with ungrouped arrays in single task. Depriving attention during estimation of ungrouped arrays, on the other hand, did not affect estimation thresholds. Given that the numerosities tested were the same across the grouped and ungrouped conditions (in both cases well exceeding the subitizing range), the only factor driving the attentional modulation was the spatial configuration. We presume that ungrouped arrays were judged primarily by estimation system, largely independently of attention, whereas grouped arrays trigger the additional intervention of the subitizing system, which boosts performance. However, as subitizing requires attentional resources, during dual-task only the estimation system could operate, bringing performance for grouped arrays down to that of ungrouped stimuli. In the grouped condition, the detrimental effect of dual task scaled both with total numerosity and with the number of groups, with stronger cost for low numerosities and lower number of groups. The higher cost of attention for low numerosities and fewer groups suggests that groupitizing acts on both these factors. With larger total numerosities and/or number of groups, the attentional free estimation system is likely to kick in, even if items are spatially segregated, resulting in a weaker attentional modulation of estimation thresholds.

We also found that estimation biases differed across attentional and grouping conditions. All estimates departed from linearity and tended toward the center of the numerosity range, with the effect increasing when attention was deprived. The observed compressed non-linearity was well fitted by a Bayesian model of central tendency^14,15,24,25^. This effect has been described for a wide range of stimuli^26–31^, and is thought to maximize the perceptual efficiency by exploiting contextual effects. An important prediction of the Bayesian model is that the magnitude of the non-linearity should depend on perceptual thresholds. This prediction was borne out, with a strong and significant correlation between magnitude of non-linearity and Weber fractions. And the Weber fractions predicted well the form of the non-linearity, with only one degree of freedom (strength of the prior, unchanged between conditions).

We further explored whether groupitizing may depend on the ability to make simple calculations on grouped stimuli^7–9^. The correlation between arithmetic skills and Weber fractions of grouped (but not ungrouped) stimuli, and also with the groupitizing advantage in our small sample suggests that this may be the case. We emphasize, however, that although estimation thresholds of ungrouped arrays were uncorrelated with math ability in our small sample of adults, we do not believe that this contradicts theories suggesting that an efficient Approximate Number System (ANS) may be a pre-requisite for typical development of math skills^23,32,33^. The link between ANS and math abilities is much less evident in adults than in children^18,34–36^. Many studies have reported that numerosity perception precision sharply improves during development and formal arithmetical learning^37–41^ (but see also^42–44^), while in educated adults, symbolic math abilities may be already steadily mapped into their basic non-symbolic representation, making the association less evident^18,36,45–47^. While ANS precision measured with ungrouped stimuli may be a reliable predictor of early math abilities in childhood, once the number acuity has refined and been mapped onto symbolic numbers, it could lose part of its predictive power. However, groupitizing relies less on approximate numerical estimation, but triggers calculation strategies to combine subitized subsets. This was confirmed by participant subjective reports. Although grouping strategies were never mentioned in participant instructions, when debriefed all participants reported to have used arithmetical strategies (addition and in some cases multiplication of the subgroups). Participants also reported that they had more difficulties in applying these strategies when the stimuli were ungrouped. In this condition, participants may have used a combination of different approaches, probably weakening the link with mental calculation skills.

Importantly, the efficiency of the subitizing system by itself may not to be sufficient to predict calculation skills. Previous studies found no significant correlation between subitizing capacity and math skills in children or adults^48^. Moreover, while the subitizing system is already functional as early as 2 years of age^49^, 6-year-old preschoolers cannot take advantage of groupitizing^7^. Thus, the relationship between groupitizing and arithmetic is most likely driven by using calculation skills to extend the subitizing range, rather than on the capacity to subitize. It should be mentioned, however, that exact serial counting speed has been shown to be a good marker of arithmetical abilities^50,51^, leaving open the possibility that the link between arithmetic and subitizing may emerge more clearly when slow counting is used instead of fast approximation, as in the current study. Also, a recent study on kindergarten children has suggested that subitizing may play a role in the development of symbolic number abilities, opening the possibility that the link would be stronger in the earliest developmental stages^52^.

In this study, like previous studies, we deliberately facilitated the use of grouping strategies by spatially grouping the stimuli. Other manipulations also aid grouping, such as organizing stimuli into same-coloured groups. It would be interesting to explore further what other organizations may encourage groupitizing. For example, mirror symmetry biases numerosity estimates, so symmetrical patterns appear less numerous than their asymmetric counterparts^53^. It is possible that symmetry would also facilitate grouping, leading to lower thresholds. This would be well worth exploring, together with other manipulations of shape and organization.

While the correlational results of this study should be taken with some caution, given the small number of participants, our explorative analysis should encourage future work investigating whether numerosity thresholds measured with grouped arrays (using a variety of grouping cues) may prove to be a more sensitive predictor of arithmetical abilities in adults. These studies should also explore the contribution of other domain general processes, such as attentional and working memory resources to the groupitizing advantage and their predictive role with different components of the arithmetical competence.
